# Anterior basolateral amygdala neurons comprise a remote fear memory engram

**DOI:** 10.3389/fncir.2023.1167825

**Published:** 2023-04-27

**Authors:** Robert J. Hammack, Victoria E. Fischer, Mary Ann Andrade, Glenn M. Toney

**Affiliations:** ^1^Department of Cellular and Integrative Physiology, University of Texas Health Science Center at San Antonio, San Antonio, TX, United States; ^2^Center for Biomedical Neuroscience, University of Texas Health Science Center at San Antonio, San Antonio, TX, United States; ^3^Department of Neurosurgery, University of Texas Health Science Center at San Antonio, San Antonio, TX, United States

**Keywords:** engram, fear, TRAP2, mouse, glutamatergic

## Abstract

**Introduction:**

Threatening environmental cues often generate enduring fear memories, but how these are formed and stored remains actively investigated. Recall of a recent fear memory is thought to reflect reactivation of neurons, in multiple brain regions, activated during memory formation, indicating that anatomically distributed and interconnected neuronal ensembles comprise fear memory engrams. The extent to which anatomically specific activation-reactivation engrams persist during long-term fear memory recall, however, remains largely unexplored. We hypothesized that principal neurons in the anterior basolateral amygdala (aBLA), which encode negative valence, acutely reactivate during remote fear memory recall to drive fear behavior.

**Methods:**

Using adult offspring of TRAP2 and Ai14 mice, persistent tdTomato expression was used to “TRAP” aBLA neurons that underwent Fos-activation during contextual fear conditioning (electric shocks) or context only conditioning (no shocks) (*n* = 5/group). Three weeks later, mice were re-exposed to the same context cues for remote memory recall, then sacrificed for Fos immunohistochemistry.

**Results:**

TRAPed (tdTomato +), Fos +, and reactivated (double-labeled) neuronal ensembles were larger in fear- than context-conditioned mice, with the middle sub-region and middle/caudal dorsomedial quadrants of aBLA displaying the greatest densities of all three ensemble populations. Whereas tdTomato + ensembles were dominantly glutamatergic in context and fear groups, freezing behavior during remote memory recall was not correlated with ensemble sizes in either group.

**Discussion:**

We conclude that although an aBLA-inclusive fear memory engram forms and persists at a remote time point, plasticity impacting electrophysiological responses of engram neurons, not their population size, encodes fear memory and drives behavioral manifestations of long-term fear memory recall.

## Introduction

Fear-eliciting stimuli often form robust and enduring associative memories that connect environmental cues to threatening life events. Subsequent encounters with similarly perceived threats result in more robust protective behaviors, reflecting fear memory recall. Errors in fear-association processing can yield negative-valence psychiatric outcomes, including anxiety and post-traumatic stress disorder (PTSD) (Tovote et al., 2015). Understanding the full complexity of mechanisms underlying fear memory is essential for identifying functional circuit abnormalities that promote fear-related psychiatric diseases and suggest novel therapeutic targets to restore normal fear processing.

Over the past several decades, fear memory research has largely focused on short-term memory formation, storage, and recall (Kim and Jung, 2006; Kim and Cho, 2020; Lee et al., 2021). Studies have implicated widely distributed interconnected CNS neuronal ensembles as responsible for fear-memory formation and storage (Ramirez et al., 2013; Liu et al., 2014; Roy et al., 2022). Activation of these ensembles by perceived threats results in potentiated synaptic transmission representing a persistent physical imprint known as a memory engram (Yuan et al., 2011; Miyawaki and Mizuseki, 2022).

Short-term memory recall is thought to reflect reactivation of the same neuronal ensembles that were activated originally during memory formation, with evidence indicating that even partial activation of a single neuronal ensemble can evoke fear-related behaviors, indicative of memory recall (Roy et al., 2022). Whilst anatomically stable potentiated circuit engrams may explain short-term fear memory, the extent to which they participate in long-term fear memory remains unclear.

Although long-term fear memory consolidation is thought to depend on progressive or sporadic reinforcement of short-term memory, several recent studies suggest that short-term fear memory neuronal ensembles undergo time-dependent reorganization, resulting in migration of the stored memory to a different neuronal ensemble either within the same brain region (DeNardo et al., 2019) or different brain regions altogether (Frankland and Bontempi, 2005; Do Monte et al., 2016).

The basolateral amygdala (BLA) is a brain region integral to fear memory and has been implicated both in recent and remote fear-memory recall (Maren et al., 1996; Gale et al., 2004; Kitamura et al., 2017; Liu et al., 2022). Notably, reports have described a specific short-term fear-memory engram in the BLA that is (1) activated during memory formation, (2) reactivated during memory recall, and (3) able to drive fear-like behaviors in non-fear contexts (Roy et al., 2022; Zaki et al., 2022). However, the extent to which a BLA neuronal ensemble activated during memory formation is reactivated during remote memory recall remains unsettled.

In this study, we tested the hypothesis that reactivation of a BLA fear memory ensemble is observed at a remote timepoint 3 weeks after fear conditioning. Of special importance, the BLA houses circuits contributing both to aversive and reward-seeking behavior (Hu, 2016), with negative-valence neurons responsive to fear stimuli localized specifically to the anterior BLA (aBLA) (Goosens and Maren, 2001; Kim et al., 2016, 2017; Zhang et al., 2020). Focusing on the aBLA, we employed second generation Targeted Recombination in Activated Populations (TRAP2) transgenic mice (DeNardo et al., 2019), in which Cre-recombinase activity is tied to *c-fos* immediate early gene promoter enhancer elements by way of an improved Cre-estrogen receptor (ER) complex. Neurons expressing this complex can undergo persistent Cre recombination, i.e., can be “TRAPed,” by administration of the ER agonist tamoxifen or its shorter-acting analog 4-hydroxytamoxifen (4-OHT), allowing for persistent expression of Cre-dependent effector or reporter molecules. Here, we crossed TRAP2 mice and the Cre-dependent tdTomato reporter line Ai14 (Madisen et al., 2010) and quantified the extent to which aBLA neurons TRAPed (tdTomato-positive) during contextual fear conditioning are reactivated (express Fos immunoreactive protein) during remote memory recall 3 weeks later. As in short-term fear memory, our findings support the presence of long-term glutamatergic fear memory neuronal ensembles in the aBLA. Notably, however, the size of aBLA neuronal ensembles, whether activated during conditioning or remote memory recall or both, was not correlated with fear behavior, indicating that the nature of fear learning-associated plasticity and its eventual impact on remote fear memory recall-associated electrophysiological responses of ensemble neurons, not the size of the ensemble population, primarily drives remote fear memory behavior.

## Materials and methods

### Ethical approval

Experimental procedures were approved by the Institutional Animal Care and Use Committee of University of Texas Health San Antonio and conformed to National Research Council *Guide for the Care and Use of Laboratory Animals.*

### Animals

**FIGURE 1:**
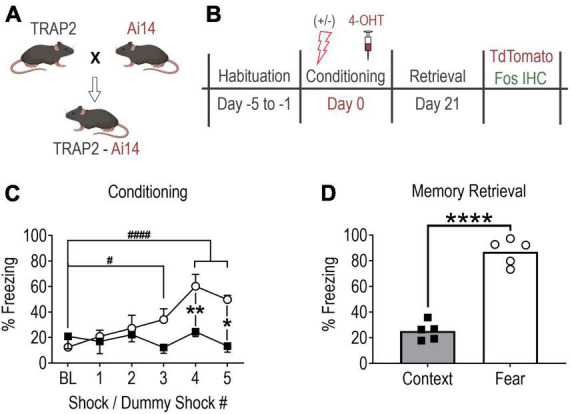
Contextual fear memory formation promotes robust remote fear memory. **(A)** Mouse breeding strategy. **(B)** Experimental protocol. Note, all mice received 4-OHT (100 mg/kg, i.p.) immediately after conditioning to “TRAP” (tdTomato) the conditioning activated fos BLA neuronal ensemble and were perfused 90 min following memory recall testing to capture the recall-activated neuronal ensemble using Fos immunohistochemistry (IHC). **(C)** Fear conditioned mice (shocks, open circles, *n* = 5) showed significant fear learning relative to baseline and relative to mice exposed to context only (no shocks, filled squares, *n* = 5). Values are the average percent of time spent freezing during a 30 s period immediately after each shock or dummy shock (^#^*P* = 0.0203, ^####^*P* < 0.0001 vs. shock group baseline (BL); **P = 0.0056, *P = 0.0044 between groups, two-way ANOVA with Sidak’s post-test. **(D)** Remote memory recall at day 21 post-conditioning (****P < 0.0001, unpaired *t*-test). Data are mean ± SEM. Images of mice created with Biorender.com.

Breeding pairs of homozygous Fos^2*A–iCreER/*2*A–iCreER*^ knock-in (TRAP2, #030323) and R26^*Ai*14/+^ (Ai14, #007914) mice (Jackson Laboratories) were crossed to produce male hemizygous TRAP2:Ai14 offspring (Figure 1A). Post weaning, mice were group housed in plastic cages (29 × 18 × 13 cm) containing rodent bedding (Sani-chips; Harlan Teklad, Madison, WI, USA). The vivarium was temperature-controlled (24°C) with a 14:10 h light-dark cycle (lights on at 0600 h). Food and water were available *ad libitum*. Experiments were initiated when mice reached 3 months old.

### Behavioral habituation

As in previously published studies (DeNardo et al., 2019), mice underwent five consecutive days of habituation training immediately prior to contextual fear conditioning to minimize *c-fos* induction in response to experimental conditions not under investigation (e.g., handling, novel context exploration, etc.) (Figure 1B). Each daily habituation session lasted 3 min and consisted of exposure to the conditioning chamber (Habitest Modular System, Coulbourn Instruments). Chamber sensory cues consisted of (1) 70% ethanol (olfactory cue), (2) metal grid floor (haptic cue), (3) patterned background (visual cue), and (4) visible light (visual cue). Additionally, all mice were hand-held for one min before being placed in the conditioning chamber and were gently “scruffed” for 10 s upon exiting the chamber. Habituation sessions were designed to mimic handling that mice would experience on the day of conditioning except no intraperitoneal (IP) injections were performed.

### Contextual fear conditioning

Mice were next randomly assigned to fear-conditioned (shock) and context-conditioned (no-shock/context-only) groups. Conditioning commenced on Day 0 and consisted of mice being placed in the conditioning chamber and allowed to explore for 2 min. Over the subsequent 4 min, fear-conditioned mice received a series of five foot-shocks of 1 s duration and 0.75 mA intensity. Shocks were delivered at intervals unpredictable to the mice, yet consistent between mice. Context-conditioned mice underwent an identical protocol excluding foot-shocks (Figure 1C). Upon task termination, each mouse received an injection of 4-hydroxytamoxifen (4-OHT, 100 mg/kg, IP) dissolved in a 4:1 cocktail of sunflower and castor oil prepared as previously described (DeNardo et al., 2019). Mice were then returned to their home cages where they were left undisturbed in the room adjacent to the behavior suite for at least 72 h prior to resuming normal housing.

Three weeks after fear conditioning (Day 21), mice underwent remote memory recall testing (Figure 1D) consisting of re-exposure to the test chamber with sensory cues identical to those present during conditioning. Mice were allowed to explore the chamber for 5 min during which fear behavior (postural freezing) was scored using FreezeFrame 4 video tracking software (ActiMetrics, Wilmette IL, USA).

### Brain fixation and histology

Ninety minutes following memory recall testing, mice were deeply anesthetized with isoflurane (5% in oxygen) then underwent transcardiac perfusion with 30 mL of heparinized (100 U/mL) isotonic saline followed by 100 mL of 4% paraformaldehyde (PFA) in 0.1 M phosphate buffer. Brains were removed, post-fixed in 4% PFA for 6 h at room temperature (∼22°C) and cryoprotected in 30% sucrose-0.01 M phosphate buffer saline (PBS) for at least 2 days. Brains were then sliced in 30 μm-thick coronal sections on a freezing microtome (Leica Microsystems, Wetzlar, Germany) and sections containing the aBLA (Paxinos and Franklin, 2019), were stored in polyvinylpyrrolidone (PVP) cryoprotectant at −20°C.

### Immunohistochemistry

Immunostaining was performed as previously described (Mitchell et al., 2018; Maruyama et al., 2019). Briefly, sections were washed in PBS to remove PVP cryoprotectant and incubated for 30 min in PBS containing sodium borohydride (0.5%) to remove auto-fluorescent aldehydes generated during fixation. Following additional PBS washes, sections were incubated in blocking buffer (3% goat serum, 0.05% Triton-X-100 in PBS) for 2 h at ∼22°C followed by incubation at 4°C in blocking buffer containing a polyclonal rabbit c-Fos primary antibody (Ab) for 72 h (1:1,500, synaptic systems #226 003) or monoclonal mouse CaMKII primary Ab for 24 h (1:500, Enzo Life Sciences, #ADI-KAM-CA002). After further washing, Fos and CaMKII sections were separately incubated for 2 h at ∼22°C in biotinylated goat anti-rabbit IgG secondary Ab (1:250 EMD Millipore #AP132B) followed by serial washing in 0.05 M tris-buffered saline (TBS) and 0.1 M sodium acetate then exposed to streptavidin-Alexa Fluor 488 (1:250, Invitrogen #S11223) for 1 h in TBS-based blocking solution. Finally, sections were washed in Tris buffer and mounted on slides with Fluoromount-G (Invitrogen, #00-4958-02).

### Imaging and analysis

A16-bit photomultiplier tube interfaced with a Zeiss LSM710 laser scanning confocal microscope, equipped with appropriate laser lines, was used to capture aBLA images using a 20× objective (NA 0.8) and a scan head pinhole size of 47.5 μm. For each image, ImageJ software (NIH, Bethesda, MD) was used to generate separate pixel maps of tdTomato (i.e., TRAPed, red) and Alexa-488 (Fos, green) fluorescence. Pixel threshold intensity and wavelength overlap detection for dual labeling was determined by comparing tissue sections processed with and without Fos primary Ab as previously described (Mitchell et al., 2018; Maruyama et al., 2019). Cell-counting and co-localization data were obtained using the semi-autonomous ImageJ plugin EzColocalization (Stauffer et al., 2018).

Images of the aBLA were divided into three rostro-caudal sub-regions of nearly equal size, with each sub-region defined by a specific range of rostral-caudal stereotaxic coordinates relative to bregma: rostral, −0.8 to −1.1 mm; middle, −1.2 to −1.5 mm; and caudal, −1.6 to −1.9 mm (Paxinos and Franklin, 2019). Region of Interest (ROI) manager in ImageJ was then used to divide the aBLA along its dorsal-ventral and medial-lateral quadrants relative to its perimeter. This template was subsequently applied to all images within each specified rostral-caudal sub-region. The aBLA perimeter demarcation was used with the ImageJ EzColocalization plug-in to generate a 2-D spatial plot of all neuronal counts in each image. Spatial plots within the rostral, middle, and caudal sub-regions were then separately overlayed. This resulted in 10 fear and 10 context plots for the rostral sub-region, 10 fear and 13 context plots for the middle sub-region, and 12 fear and 13 context plots for the caudal sub-region. To correct for a greater number of sub-region image plots in the context group, correction factors of 1.0 (10/10), 0.77 (10/13), and 0.92 (12/13) were applied, respectively, to the rostral, middle, and caudal sub-region plots of the context group. To avoid biasing the topographical distribution of counts in the context data, a random number generator in MS-Excel was used to identify which specific counts to exclude from each aBLA sub-region.

**FIGURE 2:**
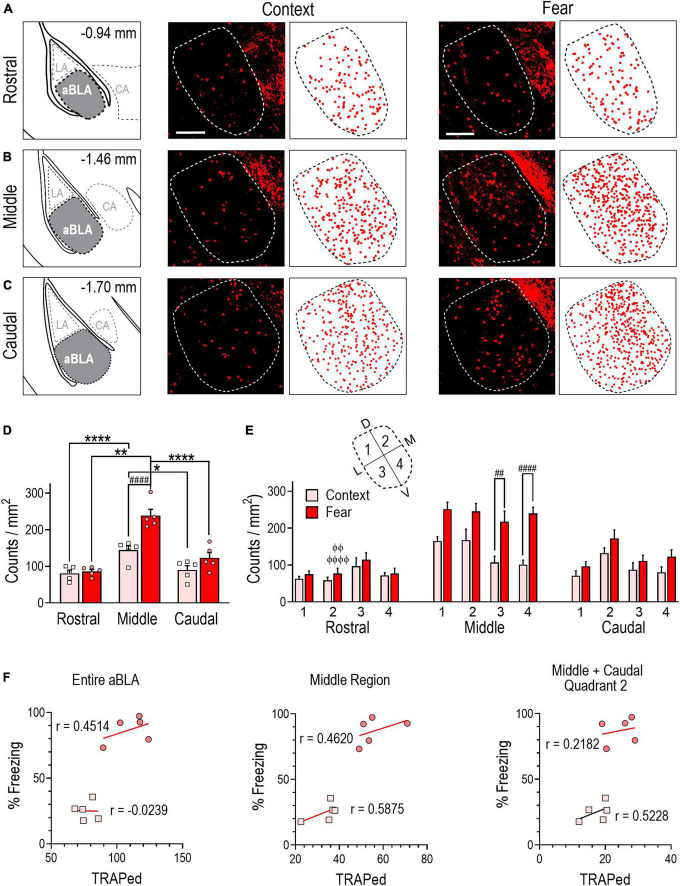
Contextual fear memory formation induces an enlarged neuronal ensemble in the aBLA. **(A–C)** Stereotaxic plate drawings of aBLA sub-regions (left), representative images of context-TRAPed tdTomato + (red) neurons and their summary distribution plot (center, *n* = 5 mice), and representative fear-TRAPed tdTomato + (red) neurons and their summary distribution plot (right, *n* = 5 mice). **(A)** Rostral sub-region. **(B)** Middle sub-region. **(C)** Caudal subregion. Scale bar (top, left) = 200 μm. **(D)** Average density of TRAPed neurons in each analyzed aBLA sub-region in context (*n* = 5) and fear (*n* = 5) groups. *P = 0.0132, ****P < 0.0001 between sub-regions in the context group. **P = 0.0035, ****P < 0.0001 between sub-regions in the fear group. ^####^*P* < 0.0001 between groups. Two-way ANOVA with Sidak’s post-test. **(E)** Distribution of TRAPed neurons in quadrants (inset) of the rostral, middle, and caudal aBLA in context and fear groups. ^##^*P* = 0.0031, ^####^*P* < 0.0001 between groups. ^ϕϕ^*P* = 0.0040, ^ϕϕϕϕ^*P* < 0.0001 between sub-region and within-fear and within-quadrant. Three-way ANOVA with Tukey’s post-test. Data are mean ± SEM. **(F)** Correlations showing the relationship between the amount of time mice spent freezing during remote context memory (squares) or fear memory (circles) recall and tdTomato + counts (TRAPed) in the entire aBLA (left), its middle sub-region (center) and in quadrant 2 of the middle and caudal sub-regions (right). In each graph, lines represent the least squares regression for each correlation. Within each treatment group, Pearson’s coefficients (r) were not significant for any correlation. Between groups, Fisher’s z transformation was applied and coefficients (r’) were not different between groups for any correlation (see Supplementary Table 1).

Next, we further divided aBLA sub-regions into quadrants, designated 1–4 (see Figure 2D). To do so, the intersection coordinate (0,0) was determined in MS-Excel as the point in the 2-D plane at which the area of each quadrant was approximately equal to one-fourth of the total area of each rostral-caudal sub-region. Quadrant axes and the x-y coordinates of every count were determined and rotated 30° clockwise, thereby aligning them to the anatomical axis of the aBLA. To account for small variations in the size of sub-regions and their quadrants, count data were normalized to surface area (counts/mm^2^) so that reported values represent the density of counts per sub-region or quadrant.

To assess the excitatory phenotype of neurons captured by our TRAP2 system, co-localization of conditioning-TRAPed tdTomato expression with calcium–calmodulin-dependent protein kinase II (CaMKII), an established marker of glutamatergic neurons (McDonald et al., 2002), was quantified from images of at least three aBLA sections per mouse using the ImageJ cell counter plug-in.

### Statistics

Statistical testing was performed with Prism 9.5.1 software (GraphPad, San Diego, CA, USA) and included testing for normal distribution of all datasets using a Shapiro-Wilk test. Group comparisons for continuous data were made with two-tailed unpaired student’s *t*-tests or by two- or three-way ANOVA followed by Sidak’s and Tukey’s multiple comparison test, respectively, for pair-wise comparisons following significant ANOVA interactions. Within-group correlation analysis was performed to determine a Pearson’s r coefficient and corresponding *P*-value. Correlations were compared across groups using Fisher’s Z transformation (Supplementary Table 1). Group data are expressed as mean ± SEM. Statistical significance was set at *P* < 0.05.

## Results

### Fear conditioning induces a remote fear-memory engram

To establish the extent to which aBLA neurons activated during fear memory formation are reactivated during remote memory recall, mice underwent fear (*n* = 5), or context (*n* = 5) conditioning followed by injection of 4-OHT (Figures 1A–C). Examining fear memory formation (i.e., fear learning), a significant interaction of treatment (shock vs. no shock) and time bin (30 s interval after each shock) on freezing behavior was observed [two-way ANOVA, *F*(5,40) = 6.733, *P* = 0.0001]. Fear-conditioned mice showed increased freezing time relative to baseline after shock 3 (275 ± 68% of BL, *P* = 0.0203), 4 (486 ± 76% of BL, *P* < 0.0001) and 5 (402 ± 26% of BL, *P* < 0.0001), and relative to context-conditioned mice after shock 4 (251 ± 38% of context, *p* < 0.0001) and 5 (375 ± 25% of context, *p* < 0.0001). As expected, re-exposure to the conditioning chamber for remote memory recall on day 21 post conditioning revealed fear-conditioned mice exhibited significantly more time spent freezing compared to context controls [346 ± 18%, unpaired *t*-test, *t*(8) = 11.16, *P* < 0.0001] (Figure 1D). Collectively, data in Figure 1 indicate that our fear conditioning protocol caused robust fear memory formation and remote fear memory recall.

### Fear learning induces a robust sub-region specific aBLA fos ensemble

Analysis of TRAPed (tdTomato +) counts revealed a significantly larger fos-activated aBLA population in fear-conditioned (110 ± 6.2) than context-conditioned (77 ± 3.1) mice [unpaired *t* test, *t*(8) = 4.782, *P* = 0.0014] (Table 1). As expected, ∼70% of context- and ∼80% of fear-TRAPed neurons (*n* = 6 aBLA sections/group) were CaMKII + (Supplementary Figures 1A, B), indicating they were primarily glutamatergic projection neurons.

**TABLE 1:** Comparative size of aBLA Fos ensembles.

Group	N	TRAPed counts (conditioning)	Fos + counts (Recall)	Reactivated Counts (TRAPed + Fos +)	% TRAPed neurons reactivated
Context	5	77 ± 3.1	83 ± 2.5	17 ± 1.8	20 ± 2.6
Fear	5	110 ± 6.2**	128 ± 5.0****	35 ± 2.2***	30 ± 1.5*

Data are mean ± SEM. Unpaired t-test, *P = 0.0101, **P = 0.0014, ***P = 0.0002, ****P < 0.0001 vs. context.

Similar to previous reports (Kim et al., 2016), we divided the aBLA into three rostro-caudal sub-regions: rostral, middle and caudal (Figures 2A–C) and compared TRAPed count densities (counts/mm^2^) across sub-regions within and between treatment groups (Figure 2D). Two-way ANOVA revealed a significant interaction between sub-region and treatment [*F*(2,24) = 7.7099, *P* = 0.0038]. Within-group analyses revealed that TRAPed count density was greater in the middle aBLA sub-region in fear (239 ± 9) and context (144 ± 12) groups relative to the rostral (fear: 86 ± 6, *P* < 0.0001; context: 81 ± 9, *P* = 0.0035) and caudal (fear: 123 ± 15, *P* < 0.0001; context: 90 ± 12, *P* = 0.0132) sub-regions. Between-group analysis revealed TRAPed count density was significantly greater in the middle sub-region of fear conditioned mice (*P* < 0.0001).

To further delineate the location of TRAPed neurons, we subdivided rostral, middle, and caudal aBLA sub-regions into four quadrants each (Figure 2E). In the middle sub-region, a main treatment effect on count density was observed [three-way ANOVA, *F*(1,32) = 17.44, *P* = 0.0002] with higher TRAPed count density in the fear than context group in quadrants 3 (218 ± 29 vs. 107 ± 17, *P* = 0.0031), and 4 (240 ± 16 vs. 101 ± 12, *P* < 0.0001). We further evaluated within-group and within-quadrant TRAPed count densities across aBLA sub-regions (Figure 2E and Supplementary Table 2). Notably, when compared to the rostral sub-region (59 ± 8), quadrant 2 of the fear group had higher TRAPed density in both the middle (245 ± 21, *P* < 0.0001) and caudal (172 ± 23, *P* = 0.0040) sub-region.

We next evaluated the relationship between freezing behavior and the size of TRAPed neuronal ensemble in the entire aBLA, in its middle sub-region, and in quadrants of the middle and caudal sub-regions where ensemble count densities differed between context and fear conditioned groups. No significant correlations were observed in either the fear or context group (Figure 2F), suggesting that fear behavior does not reflect the size of the fear-learning fos ensemble in aBLA sub-regions.

Collectively, findings in Figure 2 indicate that fear conditioning resulted in a larger fos ensemble in the aBLA than did context conditioning. Ensemble neurons were most densely localized to the middle aBLA sub-region, but fear behavior did not correlate with ensemble population sizes.

### Remote fear memory recall induces a sub-region specific aBLA Fos ensemble

**FIGURE 3:**
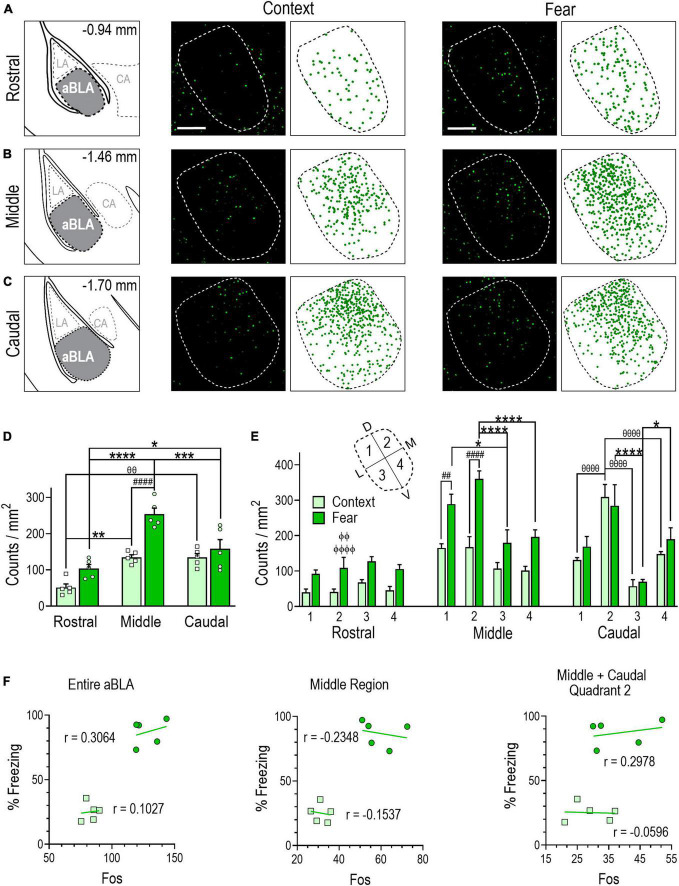
Remote contextual fear memory recall recruits an enlarged neuronal ensemble in the aBLA. **(A–C)** Stereotaxic plate drawings of aBLA sub-regions (left), representative images of Fos + (green) neurons activated during remote contextual memory recall and their summary distribution plot (center, *n* = 5 mice), and representative Fos + (green) neurons activated during remote contextual fear memory recall and their summary distribution plot (right, *n* = 5 mice). **(A)** Rostral sub-region. **(B)** Middle sub-region. **(C)** Caudal subregion. Scale bar (top, left) = 200 μm. **(D)** Average density of Fos + neurons in each analyzed aBLA sub-region in context and fear groups. **P = 0.0019, ^θθ^*P* = 0.0018 between sub-regions in the context group. *P = 0.0490, ***P = 0.0004, ****P < 0.0001 between sub-regions in the fear group. ^####^*P* < 0.0001 between groups. Two-way ANOVA with Sidak’s post-test. **(E)** Distribution of Fos + neurons in quadrants (inset) of the rostral, middle, and caudal aBLA. Middle: ^##^*P* = 0.0031, ^####^*P* < 0.0001, between groups; *P = 0.0168, ****P < 0.0001, between quadrants in the fear group. Caudal: ^θθθθ^*P* < 0.0001, between quadrants in the context group, *P = 0.0423, ****P < 0.0001, between quadrants in the fear group. ^ϕϕ^*P* = 0.0015, ^ϕϕϕϕ^*P* < 0.0001 between sub-region and within-fear and within-quadrant. Three-way ANOVA with Tukey’s post-test. Data are mean ± SEM. **(F)** Correlations showing the relationship between the amount of time mice spent freezing during remote context memory (squares) or fear memory (circles) recall and Fos + counts in the entire aBLA (left), its middle sub-region (center) and in quadrant 2 of the middle and caudal sub-regions (right). In each graph, lines represent the least squares regression for each correlation. Within each treatment group, Pearson’s coefficients (r) were not significant for any correlation. Between groups, Fisher’s z transformation was applied and coefficients (r’) were not different between groups for any correlation (see Supplementary Table 1).

Analysis of immunostained sections revealed that memory recall testing activated a significantly larger Fos + population of aBLA neurons in fear-conditioned (128 ± 5) than context-conditioned (83 ± 3) mice [unpaired *t* test, *t*(8) = 8.027, *P* < 0.0001] (Table 1). Examination of rostral-caudal aBLA sub-region count densities (Figures 3A–C) by two-way ANOVA revealed a significant sub-region and treatment interaction [*F*(2,24) = 5.402, *P* = 0.0116] (Figure 3D). In the remote fear memory recall group, aBLA Fos + count density was greater in the middle (254 ± 17) than the rostral (104 ± 11, *P* < 0.0001) or caudal (158 ± 26, *P* = 0.0004) sub-region and was greater in the caudal than rostral sub-region (*P* = 0.0490). In the context memory recall group, Fos + count density was comparable in the middle (134 ± 24) and caudal (135 ± 11) sub-regions, yet both were greater than in the rostral (51 ± 10) sub-region (middle, *P* = 0.0019; caudal, *P* = 0.0018). Between-group analyses revealed Fos + count density was greater only in the middle aBLA sub-region of the fear memory recall group (*P* < 0.0001).

Analysis of Fos + count density within quadrants by three-way ANOVA (Figure 3E) revealed quadrant [*F*(3,32) = 6.450, *P* = 0.0015] and treatment [*F*(1,32) = 78.63, *P* < 0.0001] effects within the middle aBLA sub-region, with greater Fos + count density in the fear compared to context group in quadrant 1 (289 ± 28 vs. 165 ± 12, *P* = 0.0031) and 2 (361 ± 22 vs. 167 ± 30, *P* < 0.0001). Furthermore, within-group analyses showed that fear-recall induced greater Fos + count density in quadrant 1 (289 ± 28) than quadrant 3 (180 ± 37, *P* = 0.0168), while quadrant 2 (361 ± 222) was greater than quadrants 3 (*P* < 0.0001) and 4 (197 ± 20, *P* < 0.0001). A significant quadrant effect was also observed in the caudal aBLA sub-region [*F*(3,32) = 16.21, *P* < 0.0001]. The fear-recall group had greater Fos + count density in quadrants 2 (284 ± 59, *P* < 0.0001) and 4 (190 ± 32, *P* = 0.0423) compared to quadrant 3 (70 ± 6) while the context-recall group had greater Fos + count density in quadrant 2 (309 ± 35) than quadrant 1 (132 ± 6, *P* = *P* < 0.0001), 3 (57 ± 19, *P* < 0.0001) or 4 (148 ± 7, *P* < 0.0001). Comparison of Fos + counts density within a treatment group and within quadrants across aBLA rostro-caudal sub-regions (Figure 3E and Supplementary Table 2) yielded findings similar to those of tdTomato where quadrant 2 of the middle (361 ± 22, *P* < 0.0001) and caudal (284 ± 59, *P* = 0.0015) aBLA sub-regions each had greater Fos + count density than did the rostral sub-region (109 ± 30).

We also evaluated the relationship between freezing behavior and the size of Fos + neuronal ensemble in the aBLA as a whole, in its middle sub-region and in quadrant 2 of the middle and caudal sub-regions. Again, no significant correlations were observed in either the fear or context group (Figure 3F), suggesting that fear behavior does not reflect the size of the aBLA sub-region or quadrant specific fos ensemble activated during remote fear memory recall.

Data in Figure 3 illustrate that remote fear memory recall recruited a larger aBLA Fos ensemble than context memory recall. Like the fear memory formation ensemble, the highest activation density following fear memory recall was localized to the middle sub-region and in quadrant 2 of both the middle and caudal sub-regions, but again fear behavior did not correlate with the size of these ensemble populations.

### Remote fear memory recall induces a robust sub-region specific aBLA fos reactivation ensemble

**FIGURE 4:**
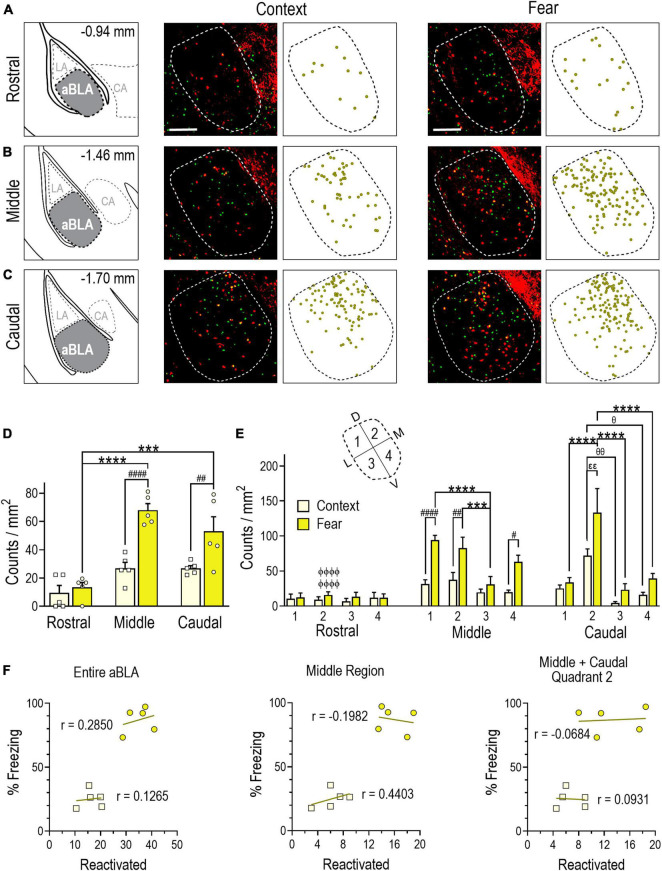
Evidence of a remote contextual fear memory engram in the aBLA. **(A–C)** Stereotaxic plate drawings of aBLA sub-regions (left), representative images of neurons reactivated + (yellow, tdTomato + and Fos +) during remote contextual memory recall and their summary distribution plot (center, *n* = 5 mice), and representative images of neurons reactivated (yellow, tdTomato + and Fos +) during remote contextual fear memory recall and their summary distribution plot (right, *n* = 5 mice). **(A)** Rostral sub-region. **(B)** Middle sub-region. **(C)** Caudal subregion. Scale bar (top, left) = 200 μm. **(D)** Average density of reactivated neurons in each analyzed aBLA sub-region in context and fear groups. ^##^*P* = 0.0098, ^####^*P* < 0.0001 between groups. ***P = 0.0001, ****P < 0.0001 between sub-regions in the fear group. Two-way ANOVA with Sidak’s post-test. **(E)** Distribution of reactivated neurons in quadrants (inset) of the rostral, middle, and caudal aBLA. Middle: ^#^*P* = 0.0129, ^##^*P* = 0.0072, ^####^*P* < 0.0001 between groups; ***P = 0.0009, ****P < 0.0001 between quadrants in the fear group. Caudal: ^εε^*P* = 0.0120 between groups; ^θ^*P* = 0.0292, ^θθ^*P* = 0.0023 between quadrants in the context group, ****P < 0.0001 between quadrants in the fear group. ^ϕϕϕϕ^*P* < 0.0001 between sub-region and within-fear and within-quadrant. Three-way ANOVA with Tukey’s post-test. Data are mean ± SEM. **(F)** Correlations showing the relationship between the amount of time mice spent freezing during remote context memory (squares) or fear memory (circles) recall and reactivation counts (tdTomato + and Fos +) in the entire aBLA (left), its middle sub-region (center) and in quadrant 2 of the middle and caudal sub-regions (right). In each graph, lines represent the least squares regression for each correlation. Within each treatment group, Pearson’s coefficients (r) were not significant for any correlation. Between groups, Fisher’s z transformation was applied and coefficients (r’) were not different between groups for any correlation (see Supplementary Table 1).

Next, we quantified neurons double labeled with tdTomato and Fos immunoreactivity (i.e., those both TRAPed during fear conditioning and reactivated during remote memory recall). Double-labeled counts in the aBLA were greater in the fear (35 ± 2) than context (17 ± 2) group [unpaired *t* test, *t*(8) = 6.505, *P* = 0.0002]. Moreover, the reactivated population was a larger proportion of the population TRAPed during fear conditioning (30 ± 2%) than context conditioning (20 ± 3%) [unpaired *t* test, *t*(8) = 3.346, *P* = 0.0101] (Table 1). Analysis of count density by two-way ANOVA yielded a significant interaction between aBLA sub-region and treatment [*F*(2,24) = 5.477, *P* = 0.0110] (Figures 4A–D). In the fear group, reactivation density was greater in the middle (*P* < 0.0001) and caudal (*P* = 0.0001) than rostral (13 ± 4) sub-region. In the context group, a similar pattern was observed in the middle (68 ± 5 vs. 27 ± 4, *P* < 0.0001) and caudal (53 ± 10 vs. 27 ± 10, *P* = 0.0098) sub-regions.

Comparing count density across quadrants of aBLA sub-regions and treatment (Figure 4E) by three-way ANOVA showed main effects for both the middle [quadrants: *F*(3,32) = 28.98, *P* < 0.0001; treatment: *F*(1,32) = 49.10, *P* < 0.0001] and caudal [quadrants: *F*(3,32) = 18.10 *P* < 0.0001; treatment: *F*(1,32) = 10.94, *P* = 0.0023] sub-regions. For the middle sub-region, remote fear memory recall induced greater neuronal reactivation compared to context in quadrant 1 (94 ± 7 vs. 31 ± 6, *P* < 0.0001), 2 (83 ± 15 vs. 38 ± 10, *P* = 0.0072), and 4 (63 ± 9 vs. 20 ± 3, *P* = 0.0129). Within the fear group, greater reactivation was also observed in quadrant 1 (*P* < 0.0001) and 2 (*P* = 0.0009) compared to 3. Within the caudal aBLA, greater reactivation (*P* = 0.0100) was observed in quadrant 2 of the fear group (133 ± 35) compared to the context group (72 ± 10). Moreover, quadrant 2 reactivation in the fear group was significantly greater than in quadrant 1 (34 ± 7, *P* < 0.0001), 3 (23 ± 9, *P* < 0.0001) and 4 (39 ± 7, *P* = 0.0002). Similarly, quadrant 2 reactivation in the context group was greater than quadrant 3 (4 ± 2, *P* = 0.0023) and 4 (16 ± 4, *P* = 0.0292). Comparison of within-group and within-quadrant reactivated count density between aBLA rostro-caudal sub-regions again yielded findings similar to tdTomato + and Fos + counts as quadrant 2 of the middle (83 ± 15, *P* < 0.0001) and caudal (133 ± 35, *P* < 0.0001) sub-regions showed greater reactivation than did the rostral sub-region (16 ± 6) (Figure 4E and Supplementary Table 2).

We next evaluated the relationship between freezing behavior and the size of the reactivated neuronal ensemble in the aBLA as a whole, in its middle sub-region and in quadrant 2 of the middle and caudal sub-regions. No significant correlations were observed in either the fear or context group (Figure 4F), suggesting that fear behavior does not reflect the size of the fos ensemble that was both activated during fear memory formation and reactivated during recall.

Figure 4 findings indicate that remote fear memory recall recruited a larger aBLA Fos reactivation ensemble than context memory recall. Like the fear memory formation and recall ensembles, the highest reactivation density was again localized to the middle sub-region and in quadrant 2 of both the middle and caudal sub-regions, but again fear behavior did not correlate with the size of these ensemble populations.

## Discussion

We used TRAP2 transgenic mice to investigate aBLA neuronal Fos ensembles activated during contextual fear learning and during remote fear memory recall. We found that larger Fos ensembles were activated by fear learning and fear memory recall compared to context only controls. The population of neurons activated both during conditioning and again during recall (reactivated) was also larger in the fear group. We also observed that ensembles were differentially distributed in aBLA sub-regions and sub-regional quadrants, but that topographical count distributions were not correlated with fear behavior.

### Fos ensemble activation during memory formation

Reports of Fos expression induced in the BLA by fear-memory testing are inconsistent (Holahan and White, 2004; Do Monte et al., 2016; Liu et al., 2022). Even after habituation training, fear conditioning with applied electrical shocks and context conditioning without shocks have both been reported to increase Fos expression compared to home cage residence (Campeau et al., 1991; Pezzone et al., 1992; Milanovic et al., 1998; Radulovic et al., 1998; Rosen et al., 1998; Day et al., 2001; Holahan and White, 2004; Cho et al., 2017), and yet Fos expression after remote fear memory recall has been reported either to increase (Silva et al., 2019; Liu et al., 2022) or not increase (Do-Monte et al., 2015; Cho et al., 2017) compared to context-recall. With fear conditioning performed after habituation training, as in the present study, Fos expressing neurons likely represent those undergoing potentiating plasticity to encode fear memory as well as those that either remain responsive to contextual cues despite habituation training or that respond to sensory inputs activated directly by the applied shocks. With context conditioning following habituation training, again as performed in the present study, Fos expressing neurons primarily represent those that remain responsive to contextual cues, some of which may undergo potentiating plasticity to encode context specific memory. There is presently no universally accepted experimental design that entirely controls for non-memory related Fos activation. Quantifying Fos expression in home cage resident controls, though potentially aiding interpretation by allowing subtraction of “background” Fos activity, does not control specifically for the direct effects of applied shocks. Moreover, neurons directly responsive to delivered shocks may not be entirely distinct from those that encode fear memory. Despite these commonly encountered design challenges, our results showing that fear memory formation and remote fear memory recall each activated a larger aBLA Fos ensemble than observed in context controls, suggests that differential Fos ensemble sizes reflect, at least partly, memory encoding due to association of negative valence unconditioned stimuli (i.e., shocks) with the contextual cues present at the time of conditioning. Differences between the current findings and some previous reports could reflect differences in the control group used (context only vs. home cage residence), intensity or timing of unconditioned stimuli (shocks), different BLA regions examined, timing of memory recall relative to conditioning (recent vs. remote) and possibly even differences in Fos staining and quantification procedures. Our use of adult TRAP2 x Ai14 offspring, however, appears unlikely to solely explain quantitative differences in Fos ensemble sizes during recall as *c-fos* transcription and translation are intact in these mice (DeNardo et al., 2019; Roy et al., 2022).

Fos protein is typically quantified by immunohistochemistry (IHC). Prior IHC studies show that Fos induced in the BLA by fear-learning peaks after ∼90 min, and remains elevated for up to 5 h (Chowdhury and Caroni, 2019). Despite the latter, Fos IHC results represent a “snapshot” of Fos activity as compared to results obtained with the TRAP2 transgenic system, which appears to capture neurons in which Fos transcription was induced over a period of ∼6 or more hours surrounding 4-OHT administration (DeNardo et al., 2019). Therefore, tdTomato expression, as an index of Fos induction, potentially reflects cumulative expression brought on during a more prolonged period. This raises the possibility that the tdTomato + TRAPed ensemble includes neurons Fos-activated by stimuli unrelated to fear/context conditioning. As noted, our use of habituation training was employed to strengthen the fear-conditioning specific signal relative to context cue- or novel environment exploration-related “noise.” With this experimental design, differential tdTomato expression may be highly relevant to understanding fear memory processes as Fos expression during hours following fear conditioning is thought necessary for memory consolidation (Chowdhury and Caroni, 2018). The TRAP2 system, therefore, might label Fos-expressing neurons that comprise a single ensemble activated specifically during fear learning *or* additional ensembles recruited in the hours thereafter, possibly including memory consolidation ensembles. Together, these considerations could explain the larger fos ensemble (tdTomato +) we detected during formation of fear memory than context memory.

### Fos ensemble during remote memory recall

#### Activation

We utilized IHC to capture the Fos ensemble (Fos +) activated during remote fear memory recall, which was larger than that captured following remote context recall. This finding is consistent with other reports of BLA Fos expression during remote fear memory recall (Silva et al., 2019; Liu et al., 2022) and parallels previous aBLA-specific reports describing increased *c-fos* mRNA expression during recent fear memory formation/recall (Kim et al., 2016; Zhang et al., 2020).

Notably, fear memory formation and remote memory recall differentially activated aBLA neurons as only about one-third underwent reactivation. Non-overlapping aBLA neuronal populations was not unexpected as previous studies indicate that a portion of BLA neurons are responsive to the sensation of the unconditioned stimulus (i.e., shocks) (Corder et al., 2019) present during fear memory formation, while another portion may be responsive specifically to conditioned stimuli (context cues) during memory recall (Beyeler et al., 2018). These variable activation patterns within aBLA ensembles during distinct fear-memory tasks (i.e., formation vs. recall) may be explained by recruitment of distinct neural circuits resulting from plasticity initiated during fear memory formation and subsequent network plasticity resulting in engram migration prior to remote memory recall testing (Grewe et al., 2017; DeNardo et al., 2019). Future *in vivo* electrophysiological studies are required to investigate responses during fear memory formation and recall, comparing across BLA fos ensemble (tdTomato +) neurons and non-fos ensemble neurons.

#### Reactivation

Reactivation of fear-learning-activated BLA Fos ensemble neurons is documented in two previous studies where remote fear memory recall took place 14 (Kitamura et al., 2017) and 28 (Lee et al., 2022) days after conditioning. While our findings at 21 days are similar, our experiments also revealed findings not previously described.

As noted, one strength of our experimental design is incorporation of a positive-control (i.e., context cue exposure only) group. This contrasts with prior remote fear-memory investigations that utilized a negative-control (i.e., home-cage) group. The latter, although permitting evaluation of basal BLA fos activity, can limit interpretation by precluding comparison of the reactivation ensemble size between context- and fear-conditioned groups (Lee et al., 2022). Our design enables this comparison and revealed significantly greater reactivation during fear-memory recall than during re-exploration of the context-conditioned environment.

Additionally, a key finding of our experiments was identification of aBLA sub-regional localization of the reactivated (engram) ensemble. Specifically, we found higher ensemble densities in dorsal zones (quadrants 1 and 2) of the middle aBLA sub-region and in the dorsomedial zone (quadrant 2) of the caudal sub-region. Prior fear memory studies have identified input and output projection neurons near the dorsomedial zone of BLA that may contribute both to memory and valence processing (Kim et al., 2016; Beyeler et al., 2018). Implicated are reciprocal connections between dorsomedial BLA and the prelimbic area of medial prefrontal cortex (PL/PFC) (McGarry and Carter, 2017) and the CA1 region of ventral hippocampus (vCA1) (Jimenez et al., 2020; Kim and Cho, 2020). These connections appear to represent synaptic substrates driving regional and sub-regional Fos induction amongst our reactivated ensembles. Another aBLA output worth noting is the capsular part of the central amygdala (Kim et al., 2016, 2017), which is responsive to noxious inputs and implicated in anxiety and fear behaviors (Bourgeais et al., 2001).

Unclear is the extent of functional heterogeneity within identified aBLA Fos ensembles. While prior studies that focused on vCA1 inputs to BLA (Kim and Cho, 2020; Lee et al., 2022) identified LTP-like synaptic potentiation amongst apparent learning ensemble neurons, it is difficult to precisely compare the location of these potentiated neurons relative to our aBLA Fos ensemble neurons. Assessing the extent to which plasticity amongst previously identified BLA neurons implicated in shorter-term contextual fear memory (Kim and Cho, 2020; Lee et al., 2022) is present among sub-regional aBLA Fos ensemble neurons of the present study and assessing their contribute to remote fear memory-related behaviors will require more detailed characterization of their neurochemical phenotypes and anatomical connectivity.

### Fos activation as an index of behavior

Here, correlation analysis failed to reveal a relationship between the size of any aBLA Fos ensemble and fear-related behavior (i.e., postural freezing), suggesting either that Fos ensemble size alone is not the primary driver of fear behavior or that the Fos ensemble is itself a representation of the memory engram and not a neuronal population directly contributing to behaviors incited by memory recall.

Within the entire aBLA (see Table 1), larger Fos ensembles (i.e., TRAPed, Fos +, and reactivated neurons) were observed in the fear group compared to the context group. A similar pattern was observed in the middle aBLA sub-region (see Figures 2D, 4D). These size differences may represent a threshold level of aBLA populational recruitment required for eliciting fear-related behaviors or it could indicate that fear behaviors require the recruitment of additional circuits. Notably, ensemble size differences between context and fear groups disappeared for some aBLA quadrants which may suggest that fear behaviors require more anatomically dispersed recruitment of aBLA neurons. Furthermore, studies show that positive valence posterior BLA (pBLA) and negative valence aBLA neurons are mutually inhibitory through activation of BLA GABAergic interneurons (Kim et al., 2016; Zhang et al., 2020). Therefore, fear behavior might reflect excitation of negative valence aBLA neurons that not only drive fear-specific outputs broadly throughout the limbic system, but also indirect inhibition of positive valence neurons of the pBLA. The latter possibility must be reconciled with evidence that individual principal neurons in the aBLA and pBLA respond both to negative and positive valence stimuli (Beyeler et al., 2018), indicating that valence and related memory encoding in BLA may not be fully segregated.

It should be stressed that the number of neurons expressing a detectable level of Fos protein is not a measure of ensemble function. What is known about the BLA fear-formation ensemble is it receives monosynaptic input from corresponding ensembles in the PL/PFC and vCA1 region of hippocampus (Kim and Cho, 2020), and at remote timepoints these inputs can both drive fear-like behaviors in a non-fear associated context (Lee et al., 2022) and restrain fear-like behaviors in a fear-associated context (Kitamura et al., 2017). However, since PL/PFC fear ensemble inputs do no exclusively synapse on BLA fear ensemble neurons (Lee et al., 2022), it is unclear to what extent differential fear behaviors reflect BLA fear ensemble activity. Therefore, future studies will not only need to delineate if the aBLA fear-formation ensemble has the capacity to influence fear-like behavior but also the capacity of fear-memory activated/reactivated ensembles to do so as well.

## Conclusion

Here we demonstrated that less than half of Fos ensemble neurons activated during memory formation are reactivated during remote memory recall, and yet fear ensembles are larger than their context counterparts specifically in the middle sub-region of aBLA and in its dorsomedial zone more caudally. Collectively, findings suggest that the remote contextual fear memory engram includes ensemble neurons of the aBLA with a common population activated during fear learning and reactivated during fear memory recall. The latter population may represent a critical sub-regional aBLA substrate through which learned fear is stored for recall at a remote time point. Maladaptive plasticity amongst these and other functionally coupled neuron populations may be key to fear-associated psychiatric disorders.

## Data availability statement

The original contributions presented in this study are included in the article/Supplementary material. Further inquiries can be directed to the corresponding author.

## Ethics statement

This animal study was reviewed and approved by the Animal Care and Use Committee, University of Texas Health Science Center at San Antonio.

## Author contributions

RH and GT designed the research. RH and MA performed the research. RH, VF, and GT analyzed the data and wrote the manuscript. All authors contributed to the article and approved the submitted version.

## Supplementary material

Supplementary Figure 1 Neurons comprising the aBLA memory formation ensemble were dominantly glutamatergic. **(A)** Images of aBLA neurons TRAPed (tdTomato +, left) in a representative context-conditioned (top) and fear-conditioned (middle) mouse together with CaMKII immunoreactivity (green) in the same section (center). Merged images (right) show TRAPed neurons that colocalized CaMKII (yellow). Scale bar (top, left) represents 30 μm. Filled white arrows indicate non-CaMKII + TRAPed neurons (not glutamatergic), open blue arrows indicate CaMKII + neurons (glutamatergic), filled yellow arrows indicate TRAPed neurons that are CaMKII + (glutamatergic memory formation ensemble neurons). **(B)** Percentage of TRAPed neurons that were CaMKII + in context and fear conditioned mice (*n* = 6/group). Co-localization counts were not different [unpaired *t*-test, *t*(10) = 1.386, *P* = 0.1959] nor were their relative proportions [Chi-square = 3.866(5), *P* = 0.5688]. Data are mean ± SEM.
