# Supplementary material for: Anterior basolateral amygdala neurons comprise a remote fear memory engram
Source: Front Neural Circuits. 2023 Apr 27;17:1167825. doi: 10.3389/fncir.2023.1167825 (PMC10174320; doi:10.3389/fncir.2023.1167825)
Supplement: Supplementary file 1 [file Table_1.pdf]

Supplemental Table 1: Pearson's r coefficient of correlation within groups and Fischer's Z comparison of correlations between groups

X	Y	Group	Pearson's r	P value	Fischer's Z	P value
			**Entire aBLA**			
TdTomato	Fos	Context	0.1526	0.8420		
TdTomato	Fos	Fear	0.6183	0.2663	0.5684	.5697
TdTomato	Memory	Context	-0.0239	0.9695		
TdTomato	Memory	Fear	0.4514	0.4454	0.5104	.6097
Fos	Memory	Context	0.1027	0.8695		
Fos	Memory	Fear	0.3064	0.6160	0.2135	.8309
Reactivated	Memory	Context	0.1265	0.8393		
Reactivated	Memory	Fear	0.2850	0.6421	0.1659	.8682
			**Middle region**			
TdTomato	Fos	Context	-0.3610	0.5506		
TdTomato	Fos	Fear	-0.5231	0.3658	0.2025	.8395
TdTomato	Memory	Context	0.4620	0.4334		
TdTomato	Memory	Fear	0.5875	0.2976	0.1740	.8619
Fos	Memory	Context	-0.1537	0.8050		
Fos	Memory	Fear	-0.2348	0.7039	0.0843	.9328
Reactivated	Memory	Context	0.4403	0.4580		
Reactivated	Memory	Fear	-0.1982	0.7493	0.6734	.5006
			**Middle + Caudal (Quadrant 2)**			
TdTomato	Fos	Context	0.6968	0.1911		
TdTomato	Fos	Fear	0.8063	0.0993	0.2553	.7984
TdTomato	Memory	Context	0.2182	0.7244		
TdTomato	Memory	Fear	0.5228	0.3661	0.3584	.7200
Fos	Memory	Context	-0.0596	0.9241		
Fos	Memory	Fear	0.2978	0.6265	0.3667	.7138
Reactivated	Memory	Context	-0.0684	0.9130		
Reactivated	Memory	Fear	0.09314	0.8816	0.16193	.8713

Fischer's z value refers to a Fischer r-to-z transformation comparing the correlations of context and fear.
