# Supplementary material for: Anterior basolateral amygdala neurons comprise a remote fear memory engram
Source: Front Neural Circuits. 2023 Apr 27;17:1167825. doi: 10.3389/fncir.2023.1167825 (PMC10174320; doi:10.3389/fncir.2023.1167825)
Supplement: Supplementary file 2 [file Table_2.pdf]

Supplemental Table 2: Within treatment group comparison of quadrant-specific ensemble densities across aBLA sub-regions

Ensemble Type	Rostral vs. Middle		Middle vs. Caudal		Rostral vs. Caudal	
**Learning (TdTomato+)**	**Context**	**Fear**	**Context**	**Fear**	**Context**	**Fear**
**Quadrant**	*F(3,32)= 0.7257, P=0.5542*		*F(3,32)= 0.5264, P=0.6633*		*F(3,32)= 0.2270, P=0.8769*	
**1 vs. 1**	0.0382	**<0.0001**	0.5740	**<0.0001**	>0.9999	0.9992
**2 vs. 2**	*0.0218*	**<0.0001**	0.9886	0.2954	0.0620	**0.0040**
**3 vs. 3**	>0.9999	**0.0357**	>0.9999	**0.0173**	>0.9999	>0.9999
**4 vs. 4**	0.9988	**<0.0001**	>0.9999	**0.0055**	>0.9999	0.6535
**Recall (Fos+)**	**Context**	**Fear**	**Context**	**Fear**	**Context**	**Fear**
**Quadrant**	F(3,32)= 1.706, P=0.1855		F(3,32)= 1.889, P=0.1514		F(3,32)= 0.5406, P=0.6580	
**1 vs. 1**	**0.0052**	**<0.0001**	>0.9999	0.2137	0.7870	0.6837
**2 vs. 2**	**0.0046**	**<0.0001**	0.1270	0.8485	**<0.0001**	**0.0015**
**3 vs. 3**	>0.9999	0.8286	>0.9999	0.3351	>0.9999	0.9420
**4 vs. 4**	0.9953	0.0932	>0.9999	>0.9999	0.5198	0.5445
**Reactivated (Dual-Labelled**	**Context**	**Fear**	**Context**	**Fear**	**Context**	**Fear**
**Quadrant**	F(3,32)= 2.454, P=0.0811		F(3,32)= 1.444, P=0.2483		F(3,32)= 0.8845, P=0.4596	
**1 vs. 1**	0.8137	**<0.0001**	>0.9999	0.1407	0.9998	0.9991
**2 vs. 2**	0.3581	**<0.0001**	0.8826	0.3799	**0.0290**	**<0.0001**
**3 vs. 3**	0.9959	0.9323	>0.9999	>0.9999	>0.9999	>0.9999
**4 vs. 4**	>0.9999	**0.0025**	>0.9999	0.9936	>0.9999	>0.9999

Comparisons of count density within a quadrant are within treatment groups and across specified sub-regions. Data represent P-values from Tukey's multiple comparison test following three-way ANOVA. Bold represents P value <0.05.
