# Supplementary figures and images for: Anterior basolateral amygdala neurons comprise a remote fear memory engram
Source: Front Neural Circuits. 2023 Apr 27;17:1167825. doi: 10.3389/fncir.2023.1167825 (PMC10174320; doi:10.3389/fncir.2023.1167825)

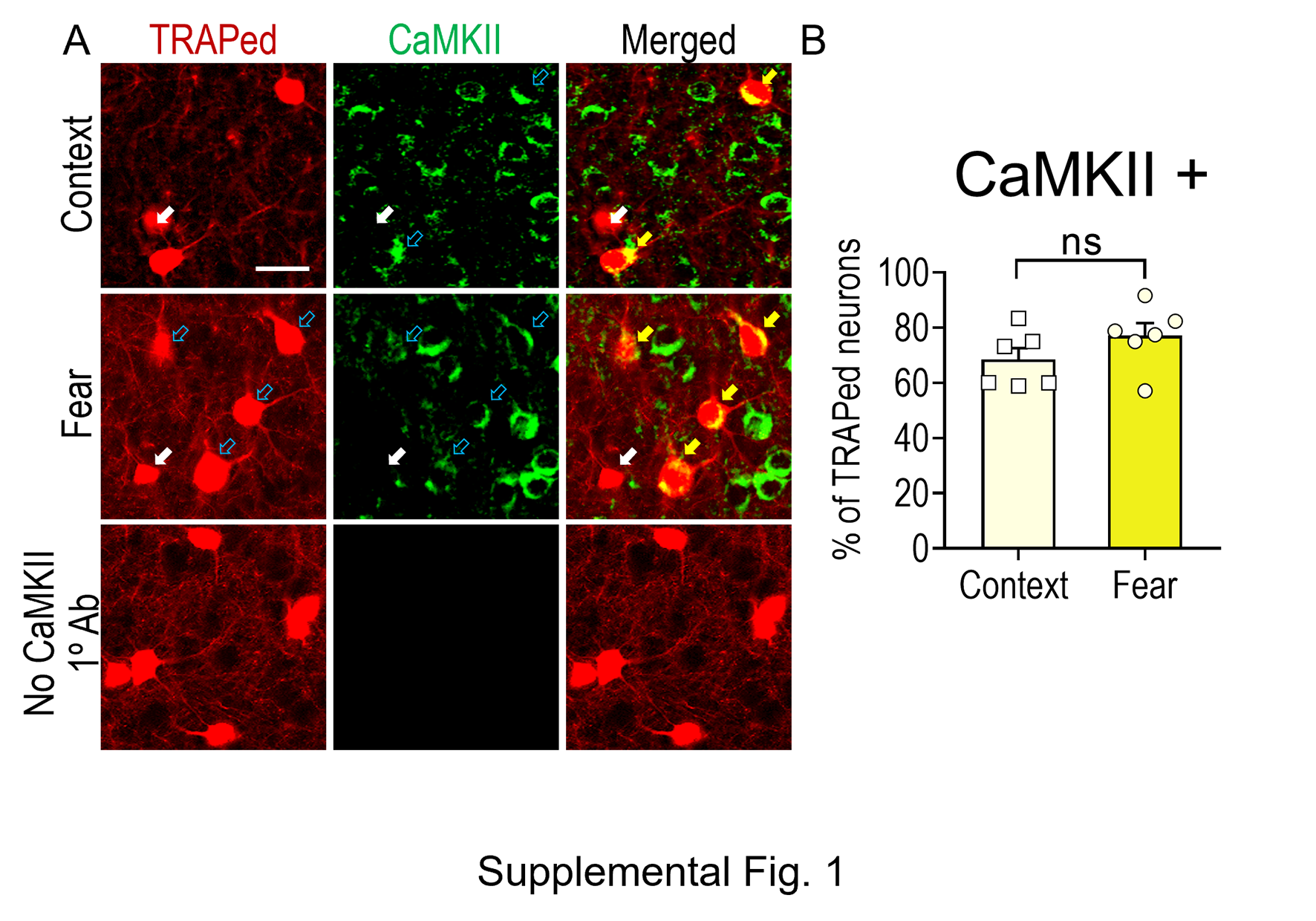

Supplement: Supplementary Figure 1 — Neurons comprising the aBLA memory formation ensemble were dominantly glutamatergic. (A) Images of aBLA neurons TRAPed (tdTomato +, left) in a representative context-conditioned (top) and fear-conditioned (middle) mouse together with CaMKII immunoreactivity (green) in the same section (center). Merged images (right) show TRAPed neurons that colocalized CaMKII (yellow). Scale bar (top, left) represents 30 μm. Filled white arrows indicate non-CaMKII + TRAPed neurons (not glutamatergic), open blue arrows indicate CaMKII + neurons (glutamatergic), filled yellow arrows indicate TRAPed neurons that are CaMKII + (glutamatergic memory formation ensemble neurons). (B) Percentage of TRAPed neurons that were CaMKII + in context and fear conditioned mice (n = 6/group). Co-localization counts were not different [unpaired t-test, t(10) = 1.386, P = 0.1959] nor were their relative proportions [Chi-square = 3.866(5), P = 0.5688]. Data are mean ± SEM. [file Image_1.tif]
